# Macrophage PARP7 Alleviates Septic Cardiomyopathy by Interacting With TBK1 and Suppressing TBK1‐Driven Inflammatory Response

**DOI:** 10.1002/advs.77404

**Published:** 2026-08-24

**Authors:** Jibo Han, Lintao Wang, Xin Zhong, Shenggang Zhao, Huihui Xie, Xue Han, Liming Lin, Yiting Lyu, Ke Xu, Jiajun Xu, Xiaowen Shi

**Affiliations:** ^1^ The Key Laboratory the Second Affiliated Hospital of Jiaxing University Jiaxing Zhejiang China; ^2^ Department of Cardiology the Second Affiliated Hospital of Jiaxing University Jiaxing Zhejiang China; ^3^ Department of Cardiology Nanjing Drum Tower Hospital Clinical College of Nanjing University of Chinese Medicine Nanjing Jiangsu China; ^4^ Laboratory Animal Center Hangzhou Medical College Hangzhou Zhejiang China; ^5^ Department of Pharmacy Wenzhou Hospital of Integrated Traditional Chinese and Western Medicine Wenzhou Zhejiang China

**Keywords:** macrophage, mono‐ADP‐ribosylation, PARP7, septic cardiomyopathy, TBK1

## Abstract

Septic cardiomyopathy is a life‐threatening complication of sepsis, and an uncontrolled inflammatory response represents a key pathogenic mechanism. PARP7 negatively regulates the IFN‐I signaling pathway through a mono‐ADP‐ribosylation‐dependent interaction with TBK1. Here, through comprehensive analysis of the expression profile of the PARP family in LPS‐treated myocardial tissues, we propose that PARP7 may be associated with septic cardiomyopathy. Then, we demonstrate that PARP7 deficiency exacerbates LPS‐induced septic cardiomyopathy in vivo. Integrated single‐nucleus and single‐cell RNA sequencing analyses demonstrate that PARP7 is predominantly upregulated in macrophages in the hearts of LPS‐treated mice. Using an AAV9‐based delivery system, we further validated the cardioprotective role of macrophage‐specific PARP7 in murine models of sepsis induced by either LPS or CLP. Mechanistically, PARP7 interacts with TBK1 to mediate its ADP‐ribosylation, thereby suppressing the TBK1‐driven inflammatory response in macrophages. The snRNA‐seq and cytokine array data collectively support a critical role for PARP7 as a molecular “brake” that constrains excessive macrophage inflammation. In conclusion, this work identifies a macrophage‐specific PARP7‐TBK1 regulatory axis in septic cardiomyopathy and highlights the therapeutic potential of macrophage‐specific PARP7 overexpression.

## Graphical abstract

In septic cardiomyopathy, PARP7 directly binds TBK1 and mediates its ADP‐ribosylation, thereby repressing TBK1‐driven proinflammatory signaling in macrophages.

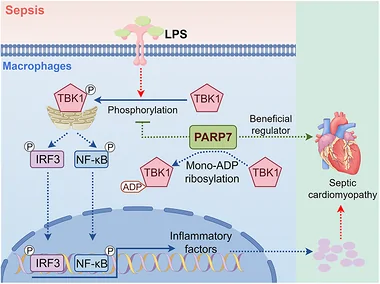

## 1 Introduction

Sepsis is an infection‐induced systemic inflammatory response syndrome characterized by multiple organ dysfunction [1]. Its hallmark is the excessive release of lipopolysaccharide (LPS), which triggers complex interactions among cytokines, immune responses, and inflammatory cells [1]. The heart is one of the organs most susceptible to sepsis, with approximately 20%–60% of sepsis patients developing myocardial dysfunction [2]. Septic cardiomyopathy is characterized by disorganization of cardiac muscle fibers, reduced contractility, and inflammatory cell infiltration, etc. Following myocardial injury, the mortality rate of sepsis can increase from 20% to 70% [2]. Although antibiotics and fluid resuscitation remain the cornerstones of current sepsis management, their efficacy is limited by the overwhelming inflammatory response [3]. Throughout all stages of sepsis, macrophage‐driven inflammatory responses play a pivotal role in disrupting cardiac homeostasis and in the pathogenesis and progression of septic cardiomyopathy [4]. Thus, from the perspective of inflammation inhibition, investigating key regulatory mechanisms and therapeutic targets in septic cardiomyopathy holds significant theoretical and clinical importance.

Poly ADP‐ribose polymerases (PARPs) are a family of 18 enzymes that transfer ADP‐ribose from nicotinamide adenine dinucleotide (NAD^+^) to target substrates, a reversible post‐translational modification known as ADP‐ribosylation [5]. Currently, inhibitors targeting PARP1 and PARP2 have been successfully applied in clinical cancer treatment, where they exert their anti‐tumor effects by impairing the DNA damage repair capacity of cancer cells, thereby inducing synthetic lethality and selective cancer cell death [6]. The clinical success of these inhibitors highlights the strong druggability of the PARPs. Furthermore, multiple PARP family members have been reported to regulate heart diseases, such as diabetic cardiomyopathy [7], doxorubicin‐induced cardiomyopathy [8], heart failure [9], and myocardial ischemia/reperfusion injury [10], through the mechanism of ADP‐ribosylation. Previously, we also investigated the pathological mechanism through which PARP1 contributes to cardiac remodeling by regulating myocardial parthanatos [11]. Therefore, PARP family members represent a promising molecular resource for identifying novel therapeutic targets in septic cardiomyopathy.

Here, through comprehensive analysis of the expression profile of the PARP family in LPS‐treated myocardial tissues, we propose that PARP7 may be associated with septic cardiomyopathy. PARP7 (also known as TIPARP) is a mono‐ADP‐ribosyl transferase that catalyzes the transfer of single ADP‐ribose units onto target substrates [12]. It contains three primary domains: a CCCH‐type zinc finger domain, a tryptophan‐tryptophan‐glutamate (WWE) domain, and a conserved PARP catalytic domain [12]. Functionally, PARP7 has emerged as an essential regulator of antiviral defense, inflammatory response modulation, and tumor suppression [13]. Currently, research on PARP7 primarily focuses on its role in negatively regulating type I interferon (IFN‐I) signaling through interaction with TANK‐binding kinase 1 (TBK1) [14]. The reported PARP7 inhibitor, RBN‐2397, is in the second phase of clinical trials and have received implied approval for clinical investigation in China, with a primary indication for the inhibition of solid tumors. However, during clinical trials, these inhibitors have been associated with adverse effects, including myocardial damage [15].

This study aims to investigate the role of PARP7 in septic cardiomyopathy and elucidate the underlying molecular mechanisms. We first demonstrate that PARP7 deficiency exacerbates LPS‐induced septic cardiomyopathy in vivo. Single‐nucleus and single‐cell RNA sequencing analyses reveal that PARP7 is predominantly upregulated in macrophages in the hearts of LPS‐treated mice. Subsequently, we validated the cardioprotective role of macrophage‐specific PARP7 in murine model of sepsis induced by either LPS or cecal ligation and puncture (CLP) using an AAV9‐based delivery system. Mechanistically, we show that PARP7 interacts with TBK1 to mediate its ADP‐ribosylation, thereby suppressing the TBK1‐driven inflammatory response in macrophages. Results from snRNA‐seq and cytokine array analyses indicate that PARP7 functions as a critical negative regulator, a molecular “brake,” in modulating macrophage inflammation. This study identifies a macrophage‐specific PARP7‐TBK1 regulatory axis in septic cardiomyopathy and highlights the therapeutic potential of macrophage‐specific PARP7 overexpression.

## 2 Materials and Methods

### 2.1 Animal Experiments

All animal experimental protocols were conducted in accordance with the Guide for the Care and Use of Laboratory Animals issued by the US National Institutes of Health and were approved by the Ethics Committee of the Second Affiliated Hospital of Jiaxing University (Approval No. JUMC2025‐187). Wild‐type C57BL/6J (WT) mice and *Parp7* knockout (*Parp7^−/−^*, Strain No. T017191) mice were obtained from GemPharmatech. The targeted disruption of the *Parp7* gene was achieved using CRISPR‐Cas9‐mediated gene editing technology. Mice were housed under specific pathogen‐free (SPF) conditions with a 12‐h light/dark cycle and provided standard rodent chow ad libitum. Male mice aged 6–8 weeks (body weight: 22–26 g) received an intraperitoneal injection of LPS (10 mg/kg, L2630, Sigma–Aldrich; 6 h) to induce septic cardiomyopathy [16]. This 6‐h LPS challenge triggers systemic inflammation and early myocardial dysfunction, while preserving sufficient cardiomyocyte viability and transcriptional responsiveness for mechanistic interrogation [17].

CLP was performed as previously described [18]. In brief, after anesthetizing mice with isoflurane, the skin was incised and the cecum carefully exposed while preserving the surrounding blood vessels. Following ligation, the distal end of the cecum was punctured and gently compressed to extrude a small amount of fecal contents, then the cecum was returned to the abdominal cavity. After wound closure, 1 mL of sterile saline was injected intraperitoneally. Sham‐operated mice underwent the same surgical procedure but without ligation or puncture. 24 h later, CLP‐induced mice exhibited ocular abscesses, reduced food intake, and decreased activity levels.

Macrophage‐specific PARP7 knockdown was achieved via systemic delivery of recombinant adeno‐associated virus serotype 9 (AAV9) encoding short hairpin RNA (shRNA) targeting the murine *Parp7* sequence (CCTTGAGCTTGTGTTTGAACTTGTA) under the control of the *F4/80* promoter. The AAV9‐F4/80‐shPARP7 vector (2 × 10^11^ viral genomes per mouse) or negative control RNA (NC) was administered by tail vein injection. Four weeks after viral delivery, septic cardiomyopathy was induced either by intraperitoneal LPS injection (10 mg/kg) or CLP.

Macrophage‐specific PARP7 overexpression was similarly achieved using AAV9 vectors expressing murine *Parp7* cDNA (NM_178892.5) under the *F4/80* promoter. Mice received either AAV9‐F4/80‐*Parp7* or control AAV9‐*F4/80*‐empty vector (EV) at 2 × 10^11^ vg/mouse via tail vein injection. Four weeks post‐injection, septic cardiomyopathy was induced by intraperitoneal LPS administration (10 mg/kg).

Cardiac function was assessed at 6 h post‐LPS administration and 24 h post‐CLP surgery using transthoracic echocardiography (Fujifilm VisualSonics). Measurements were based on the average of three consecutive cardiac cycles to ensure accuracy and reproducibility. At the conclusion of experiments, mice were euthanized under sodium pentobarbital anesthesia, followed by blood collection. Myocardial tissues were harvested for histological analysis or rapidly frozen in liquid nitrogen for subsequent molecular assays.

### 2.2 Serum Biochemical Analysis

Plasma samples were collected at 6 h post‐LPS administration and 24 h post‐CLP surgery. Serum levels of CK‐MB (E‐EL‐M0355c) and cTnT (E‐EL‐M1801c) were quantified using specific ELISA kits from Elabscience Biotechnology Co., Ltd. Absorbance was measured with the Multiskan GO Microplate Reader (Thermo Fisher Scientific) according to the manufacturer's instructions, and concentrations were calculated based on standard curves.

### 2.3 Histological and Immunohistochemical Analysis

Heart tissue sections were deparaffinized in xylene and rehydrated through a graded ethanol series. Hematoxylin and eosin (H&E; G1120, Solarbio) staining was performed to evaluate histopathological injury. Stained sections were examined under a light microscope (Zeiss).

To assess inflammatory cell infiltration, immunohistochemical staining for F4/80 and CD11b was conducted. After deparaffinization and rehydration, antigen retrieval was achieved via heat induction in citrate buffer (pH 6.0) using a thermal cycling protocol: 2 min of incubation followed by 2 min of cooling, repeated three times to maximize antigen exposure. Endogenous peroxidase activity was blocked by treatment with a peroxidase inhibitor solution. Sections were then blocked with a blocking buffer and incubated overnight at 4°C with primary antibodies against F4/80 (1:200, 70076, CST) and CD11b (1:200, ab133357, Abcam). The next day, sections were incubated with secondary antibodies for 2 h, developed with DAB substrate solution, and counterstained with hematoxylin for nuclear visualization. Immunostaining intensity was quantified as the percentage area positive per field across multiple heart tissue sections under blinded conditions. All slides were analyzed using a light microscope (Zeiss).

### 2.4 Immunofluorescence Staining

Frozen heart tissues were used to perform immunofluorescence analysis. Frozen heart tissues were used to perform immunofluorescence analysis. Briefly, sections were incubated overnight at 4°C with primary antibodies against F4/80 (HA721520, Huabio; 1:200), Flag (F1804, Millipore; 1:200), iNOS (ab49999, Abcam; 1:50), or CD206 (24595, Cell Signaling Technology; 1:100). Alex 594‐conjugated goat anti‐mouse (HA1126, HUABIO, 1:200) and iFluor 488 conjugated goat anti‐rabbit (HA1121, HUABIO, 1:200) antibodies were used as secondary antibodies. Nuclei were stained with DAPI (C0065, Solarbio). All slides were analyzed using a fluorescence microscope (Leica).

### 2.5 Single‐Cell mRNA Sequencing

Single‐cell mRNA sequencing (scRNA‐seq) was performed on myocardial tissues from LPS‐treated mice or heart failure patients using cell suspension analysis. Tissue samples were enzymatically digested in a dissociation medium containing 0.35% collagenase IV, 2 mg/mL papain, and 120 Units/mL DNase I, followed by incubation at 37°C with shaking at 100 rpm for 20 min to generate a single‐cell suspension. The digestion was terminated by adding PBS supplemented with 10% FBS, followed by repeated trituration (5–10 cycles) using a Pasteur pipette. Single‐cell suspensions were then loaded onto the MobiNova‐100 Single‐Cell System to capture approximately 5000 cells per sample, according to the manufacturer's instructions for the MobiCube Single‐Cell 3′ RNA‐seq Kit. Subsequent steps, including cDNA amplification and library construction, were carried out following the standard protocol. Libraries were sequenced on an Illumina NovaSeq 6000 platform using a paired‐end 150 bp multiplexing configuration by LC‐Bio Technology Co., Ltd. (Hangzhou, China).

### 2.6 Single‐Nucleus mRNA Sequencing

Single‐nucleus mRNA sequencing (snRNA‐seq) was performed on heart tissues from LPS‐treated WT and *Parp7^−/−^* mice, as well as from patients with heart failure, using single‐nuclei extraction analysis. Nuclei were isolated using Nuclei EZ Lysis Buffer to minimize RNA degradation. The lysis reaction was terminated by adding ice‐cold PBS containing 4% BSA, followed by centrifugation at 300 × g for 5 min at 4°C. The nuclear pellet was resuspended in lysis buffer supplemented with 4% BSA, subjected to debris removal using Miltenyi Debris Removal Solution, and washed with buffer. After an additional centrifugation step, the suspension was filtered through a cell strainer to remove aggregates and resuspended in appropriate buffer. Single‐nuclei suspensions were loaded onto the MobiNova‐100 Single‐Cell System to capture approximately 10 000 nuclei per sample according to the manufacturer's instructions for the MobiCube Single‐Cell 3′ RNA‐seq Kit. Subsequent steps, including cDNA amplification, library construction, and sequencing, were carried out following the same protocol as scRNA‐seq. Cell‐cell communication mediated by ligand‐receptor complexes was analyzed using the CellChat R package.

### 2.7 Cell Culture and Treatment

Mouse bone marrow‐derived macrophages (BMDMs) were isolated according to our previously described protocols [16]. The femurs and tibias were flushed to collect bone marrow cells. The cell suspension was filtered through a cell strainer, and red blood cells were lysed using red blood cell lysis buffer. After centrifugation, the supernatant was discarded, and the pellet was resuspended in culture medium and incubated with recombinant mouse M‐CSF. Cells were continuously supplemented with M‐CSF at a final concentration of 10 ng/mL throughout the culture period to promote differentiation into mature macrophages. NIH/3T3 cells were obtained from the Shanghai Institute of Biochemistry and Cell Biology and cultured in DMEM containing 4.5 g/L glucose, 10% FBS, and penicillin/streptomycin (100 U/mL). THP‐1 cells were purchased from the Shanghai Institute of Biochemistry and Cell Biology and cultured in RPMI‐1640 medium supplemented with 10% FBS, penicillin/streptomycin (100 U/mL), and 0.05 mm β‐mercaptoethanol. Cells were maintained in a humidified incubator at 37°C under 5% CO_2_. For in vitro stimulation experiments, LPS was applied at a concentration of 10 µg/mL. For in vitro stimulation experiments, cells were treated with LPS at 10 µg/mL, a concentration validated to activate inflammatory signaling pathways while maintaining cell viability, thereby modeling key features of sepsis‐associated macrophage activation observed in hearts.

Protein overexpression in BMDMs, NIH/3T3, and THP‐1 cells was achieved by plasmid transfection. Plasmids encoding Flag‐PARP7‐WT, △CCCH, △WWE, △PARP, H532A, and Myc‐TBK1 were provided by Genechem (Shanghai, China). Transfections were performed using Lipofectamine 3000 (L3000‐015, Invitrogen) according to the manufacturer's instructions.

To knock down TBK1 expression, small interfering RNA (siRNA) targeting TBK1 was designed (sense: 5′‐GCAGCUGUUAUCGCUAACUTT‐3′; antisense: 5′‐AGUUAGCGAUAACAGCUGCTT‐3′) and synthesized by GenePharma. siTBK1 was transfected into BMDMs using Lipofectamine 2000 (11668‐019, Invitrogen).

### 2.8 Cytokine Antibody Array

BMDMs isolated from WT or *Parp7^−/−^* mice were exposed to LPS (10 µg/mL for 24 h), and the cell supernatant was collected. Detection of cytokines in cell supernatant was performed using mouse cytokine array Q4000 (QAM‐CAA‐4000, RayBiotech). The fluorescence signal was measured using a laser scanner (InnoScan 300 Microarray Scanner, Innopsys). The raw data obtained from chip scanning were processed using RayBiotech Q‐Analyzer software to perform background subtraction and inter‐chip normalization.

### 2.9 Flow Cytometry Analysis

For cardiac flow cytometry analysis, hearts from LPS‐treated WT and *Parp7* ^−/−^ mice were harvested. Heart tissue fragments were digested with Liberase DH (Sigma 05401054001, 37°C, 30–40 min) and strained (100 µm) for single‐cell suspension. The single‐cell suspension of cardiac tissue was incubated with Fixable Viability Dye APC‐A750 (FVD) on ice in the dark for 15 min to label dead cells. After washing, cells were blocked with anti‐CD16/CD32 Fc blocker (Elabscience E‐AB‐F0997A) at 4°C for 10 min to avoid nonspecific antibody binding. Fluorochrome‐conjugated antibodies against F4/80 (111703, BioLegend), CD11b (101205, BioLegend), CD86 (105012, BioLegend), and CD206 (141719, BioLegend) were then added. Finally, cells were washed and resuspended in an appropriate volume for flow cytometry analysis by FACS Aria flow cytometer (BD Biosciences).

### 2.10 Western Blotting and Co‐Immunoprecipitation Assay

Myocardial tissues and cultured cells were lysed using cell lysis buffer (AR0101/AR0103, Boster) and protein concentrations were determined by the BCA Protein Assay Kit (P0009, Beyotime). Protein lysates were separated by SDS‐polyacrylamide gel electrophoresis and transferred onto PVDF membranes (Millipore). Membranes were blocked with 3% BSA for 2 h at room temperature to prevent non‐specific binding, followed by incubation with primary antibodies overnight at 4°C. The primary antibodies used were: PARP7 (NBP2‐93275, NOVUS, 1:1000), TBK1 (67211‐1‐Ig, Proteintech, 1:1000), P‐TBK1 (Ser172) (5483, CST, 1:1000), Flag (20543‐1‐AP, Proteintech, 1:1000), IRF3 (ET1612‐14, HUABIO, 1:1000), P‐IRF3 (Ser396) (HA722772, HUABIO, 1:1000), Mono‐ADP Ribose (89190, CST, 1:1000), NF‐κB/p65 (80979‐1‐RR, Proteintech, 1:1000), P‐NF‐κB/p65 (80379‐2‐RR, Proteintech, 1:1000), Tubulin (2128, CST, 1:1000), and GAPDH (5174, CST, 1:1000). On the following day, membranes were incubated with HRP‐conjugated goat anti‐rabbit IgG (7074, CST, 1:5000) or anti‐mouse IgG (7076, CST, 1:5000) for 2 h at room temperature. Protein bands were detected using ECL luminescence reagent (WBKLS0500, Merck Millipore), and images were visualized and analyzed using the Bio‐Rad Image Analysis System.

For co‐immunoprecipitation (Co‐IP) experiments, proteins were extracted from myocardial tissues or cultured cells using lysis buffer. An aliquot of the lysate was retained as input control. The remaining lysates were incubated with specific target antibodies or anti‐Flag magnetic beads (B26102, Bimake) overnight at 4°C. Immune complexes were captured using protein A+G agarose beads (P2055, Beyotime) with gentle rotation for 4 h at 4°C. The bead‐protein complexes were washed three times with PBS and subsequently subjected to Western blot analysis.

### 2.11 RNA Extraction Followed by Real‐Time Quantitative PCR Analysis (RT‐qPCR)

Total RNA from myocardial tissues and cultured cells was extracted by RNA‐quick purification kit (ES Science). The concentration and purity of RNA were assessed with NanoDrop 2000 Spectrophotometer (Thermo Fisher). Complementary DNA (cDNA) was synthesized using the PrimeScript RT Master Mix Kit (RR036A, Takara). Subsequently, RT‐qPCR was performed with SYBR Green Premix Ex Taq II (RR820A, Takara). Data on the target mRNA were evaluated using the 2^−ΔΔCt^ method and were normalized against the expression of *β‐actin*. Primer sequences are listed in Table S1.

### 2.12 Proximity Ligation Assay

The proximity ligation assay (PLA) was performed using a PLA kit (60025 Red, Navinci) to detect protein‐protein interactions. Cells were fixed with 4% paraformaldehyde for 15 min at room temperature, washed three times with PBS, and then permeabilized with 0.5% Triton X‐100 for 10 min. After permeabilization, cells were blocked in blocking buffer for 1 h at room temperature to minimize non‐specific binding. Primary antibodies, rabbit anti‐PARP7 (NBP2‐93275, Novus Biologicals, 1:200) and mouse anti‐TBK1 (67211‐1‐Ig, Proteintech, 1:200), were applied and incubated overnight at 4°C. Following primary antibody incubation, species‐specific PLA probes (Navenibody M1 and Navenibody M2) were added and incubated at 37°C for 60 min. Ligation and amplification reactions were carried out sequentially at 37°C for 90 min to generate detectable fluorescent signals. Nuclei were counterstained with DAPI. Fluorescent signals were visualized using a confocal laser scanning microscope (FV3000, Olympus), and images were analyzed with ImageJ software.

### 2.13 Statistical Analysis

All statistical analyses were performed using GraphPad Prism 10.0 software. Experimental data are presented as mean ± standard error of the mean (SEM). For comparisons between two groups, statistical significance was assessed using the unpaired Student's *t*‐test. For multiple group comparisons, one‐way ANOVA followed by Bonferroni's post hoc test was applied. Statistical significance was set at *p* < 0.05.

## 3 Results

### 3.1 Screening of PARP Family Expression Revealed That PARP7 may be Implicated in Septic Cardiomyopathy

To investigate the functional roles of the PARP family in septic cardiomyopathy, we first analyzed the expression profiles of PARP family members in two RNA‐seq datasets derived from myocardial tissues of LPS‐treated mice or rats. Several PARP family members exhibited upregulated expression in myocardial tissues following LPS stimulation, including *Parp9*, *Parp7*, *Parp10*, *Parp14*, *Parp12*, and *Parp3* (Figure 1A,B). We further validated these findings by examining PARP family expression in myocardial tissues of septic mice using RT‐qPCR (Figure 1C) and performed an integrated analysis across all three datasets (Figure 1D). Notably, PARP7 showed consistently increased expression across all experimental cohorts following LPS‐induced myocardial injury, implicating it as a potential regulator in septic cardiomyopathy. These observations were corroborated at the protein level, where PARP7 was significantly upregulated in LPS‐challenged myocardial tissues (Figure 1E). Here, through analysis of the expression profile of the PARP family in LPS‐treated myocardial tissues, we propose that PARP7 may be associated with septic cardiomyopathy and warrants further investigation into its functional role and underlying mechanisms.

**FIGURE 1:**
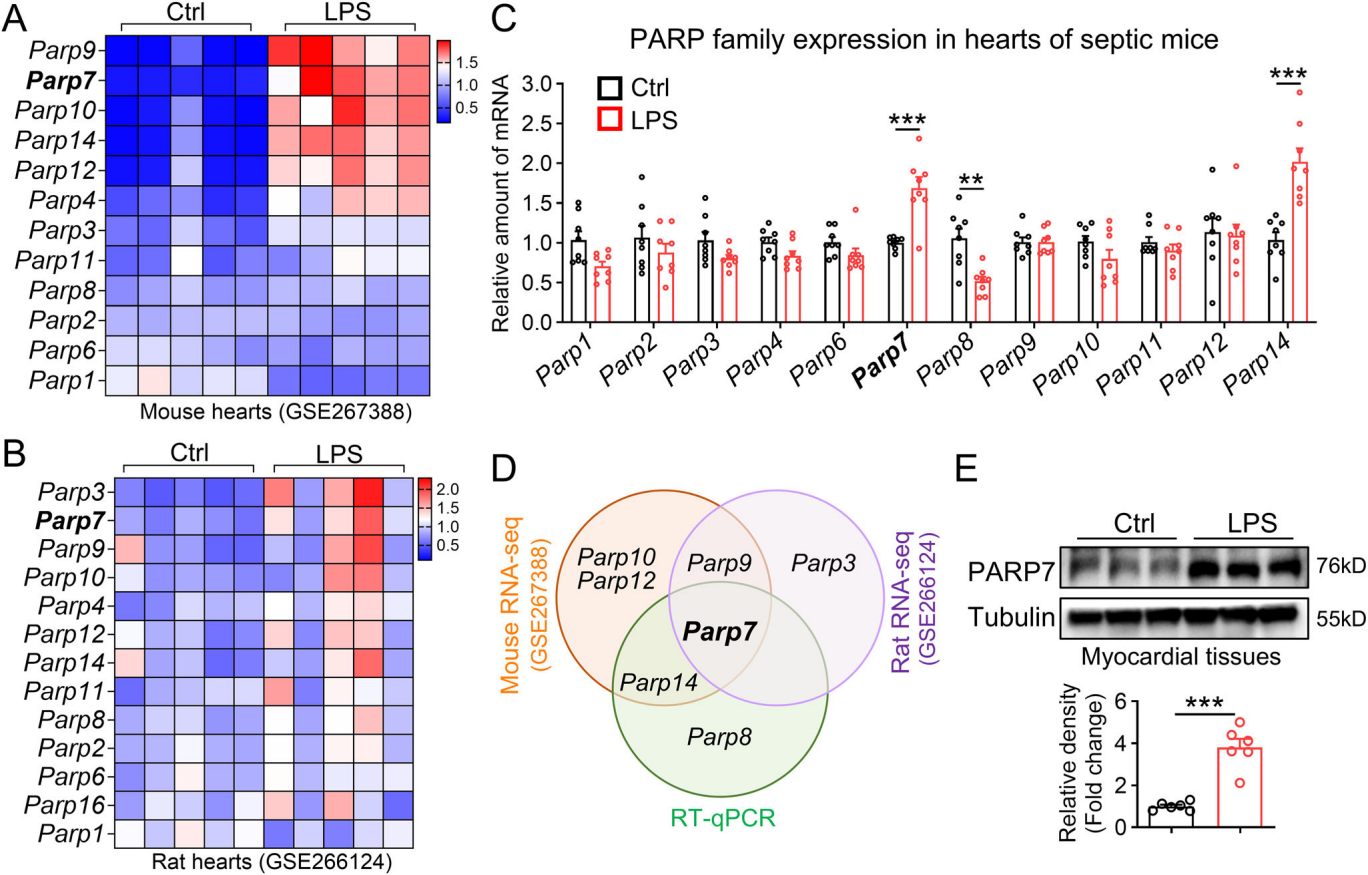
Screening of the PARP family revealed that PARP7 may be involved in septic cardiomyopathy. (A,B) The heatmap illustrates the expression profiles of PARP family genes in myocardial tissues from mouse and rat models of LPS‐induced sepsis. The RNA‐seq data presented were obtained from the GEO database (mouse: GSE267388, A; rat: GSE266124, B). (C) RT‐qPCR was used to measure the mRNA levels of PARP family members in mouse myocardial tissues following LPS treatment (10 mg/kg, 6 h, *n* = 8). (D) Integrated RNA‐seq and RT‐qPCR analyses identified PARP7 as a significantly upregulated regulator in LPS‐induced septic cardiomyopathy. (E) Western blot analysis confirmed elevated PARP7 protein levels in myocardial tissues of LPS‐treated mice (*n* = 6). Data are presented as mean ± SEM; **p < 0.01, ***p < 0.001.

### 3.2 PARP7 Deficiency Exacerbates LPS‐Induced Septic Cardiomyopathy In Vivo

We further investigated the role of PARP7 in cardiac dysfunction, histopathological injury, and inflammatory responses in LPS‐treated mice using *Parp7* knockout mice (*Parp7* ^−/−^). Genotyping was confirmed by tail DNA analysis (Figure S1A), and western blotting validated the efficient ablation of PARP7 in cardiac tissues (Figure S1B). As shown in Figure 2A, WT and *Parp7* ^−/−^ mice were administered normal saline (NS) or LPS (10 mg/kg) for 6 h. Serum levels of creatine kinase‐MB (CK‐MB, Figure 2B) and cardiac troponin T (cTnT, Figure 2C) were significantly elevated following LPS administration and further increased in *Parp7* ^−/−^ mice compared to WT controls. Consistently, echocardiographic assessment (Figure 2D) revealed that both WT and *Parp7* ^−/−^ mice exhibited marked reductions in ejection fraction (EF, Figure 2E) and fractional shortening (FS, Figure 2F) after LPS challenge, with these functional impairments being more severe in *Parp7* ^−/−^ mice (Figure 2D‐F). H&E staining of myocardial sections showed that PARP7 deficiency exacerbated LPS‐induced disorganization of cardiac muscle fibers (Figure 2G).

**FIGURE 2:**
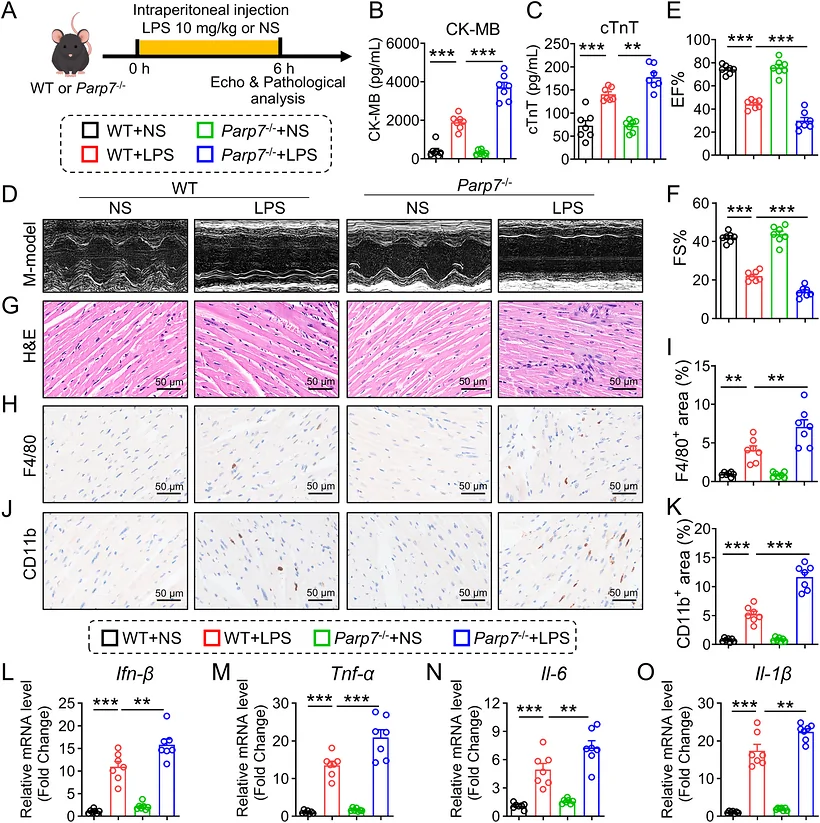
PARP7 deficiency exacerbates LPS‐induced cardiac dysfunction, histopathological damage, and inflammatory responses in mice. (A) Experimental workflow for WT or *Parp7* ^−/−^ mice administered normal saline (NS) or LPS (10 mg/kg, 6 h). Cardiac function was assessed 6 h after LPS administration. (B,C) Serum levels of creatine kinase‐MB (CK‐MB, B) and cardiac troponin T (cTnT, C) in WT and *Parp7* ^−/−^ mice following LPS treatment (*n* = 7). (D) Representative M‐mode echocardiographic images of the left ventricle. (E,F) Echocardiographic assessment of ejection fraction (EF%, E) and fractional shortening (FS%, F) in mice (*n* = 7). (G) Representative hematoxylin‐eosin (H&E) staining images. (H–K) Representative immunohistochemical staining images for F4/80 (H) and CD11b (J), along with corresponding quantitative results (I, K) (*n* = 7). (L–O) The mRNA expression levels of *Ifn‐β* (L), *Tnf‐α* (M), *Il‐6* (N), and *Il‐1β* (O) in myocardial tissues from WT and *Parp7* ^−/−^ mice treated with LPS (*n* = 7). Data are presented as mean ± SEM; **p < 0.01, ***p < 0.001.

Following sepsis, a large number of macrophages are recruited into the heart and become the predominant immune cell population [19]. These infiltrating macrophages exhibit interferon‐stimulated and pro‐inflammatory phenotypes [20]. To evaluate inflammatory cell infiltration, immunohistochemical analysis was performed on myocardial tissue sections. LPS‐treated WT mice displayed evident infiltration of F4/80‐positive (Figure 2H,I) and CD11b‐positive (Figure 2J,K) macrophages, which was markedly enhanced in *Parp7* ^−/−^ mice. Furthermore, quantitative analyses of myocardial mRNA levels revealed that the expression of pro‐inflammatory cytokines, including *Ifn‐β*, *Tnf‐α*, *Il‐6* and *Il‐1β*, was significantly upregulated by LPS and further amplified in *Parp7* ^−/−^ mice (Figure 2L–O). Collectively, these findings demonstrate that PARP7 exerts a cardioprotective effect against LPS‐induced cardiac dysfunction, histopathological damage, inflammatory cell infiltration, and cytokine overproduction.

### 3.3 Single‐Nucleus and Single‐Cell RNA Sequencing Analyses Reveal That PARP7 is Predominantly Upregulated in Macrophages During Septic Cardiomyopathy

To gain a more comprehensive understanding of the dynamic changes in PARP7 expression following LPS stimulation, we performed single‐nucleus mRNA sequencing (snRNA‐seq) in mouse hearts to examine cell‐type‐specific distribution patterns. In the snRNA‐seq analysis, we profiled 12619 nuclei from two experimental groups and identified 8 distinct cardiac cell populations: smooth muscle cells (SMCs), pericytes (PCs), NK/T cells, neuronal cells, macrophages (Macs), fibroblasts (FBs), endothelial cells (ECs), and cardiomyocytes (CMs) (Figure 3A). Notably, *Parp7* mRNA levels were upregulated in PCs, NK/T cells, neuronal cells, Macs, FBs, ECs, and CMs upon LPS challenge, with the highest distribution in macrophages (Figure 3B). To obtain a more comprehensive gene expression profile encompassing both nuclear and cytoplasmic transcripts, we performed single‐cell mRNA sequencing (scRNA‐seq). In the scRNA‐seq analysis, we profiled 21899 cells from two groups of hearts and identified seven major cardiac cell clusters: NK/T cells, B cells, SMCs, PCs, Macs, FBs, and ECs (Figure 3C). Consistent with the snRNA‐seq data, *Parp7* mRNA levels were also elevated in Macs, FBs, and ECs following LPS stimulation (Figure 3D). Notably, the LPS‐induced increase in *Parp7* was predominantly observed in macrophages (Figure 3D).

**FIGURE 3:**
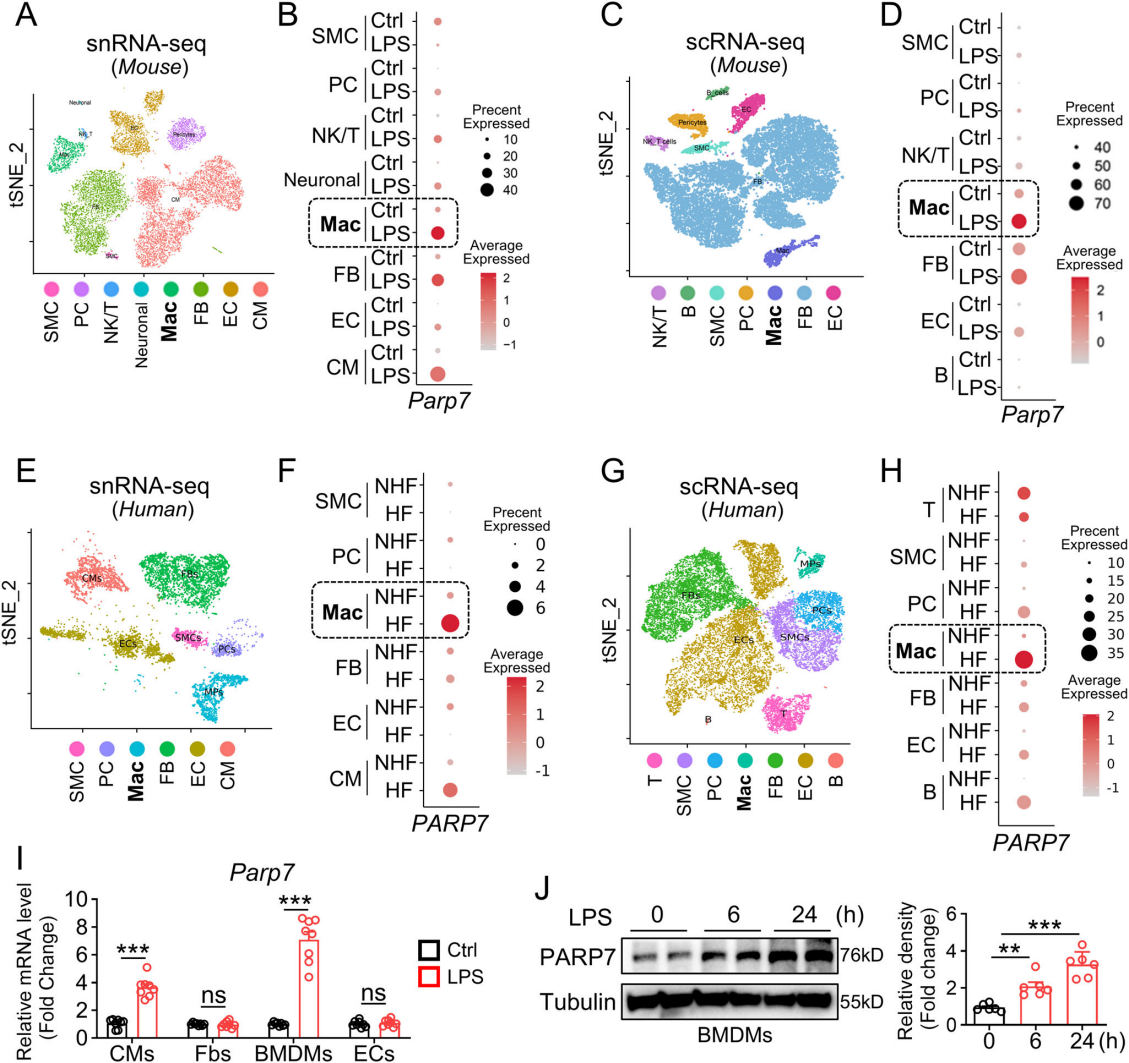
Single‐nucleus and single‐cell RNA sequencing analyses reveal that PARP7 is predominantly upregulated in macrophages during septic cardiomyopathy. (A,B) Single‐nucleus RNA sequencing (snRNA‐seq) was performed on hearts from LPS‐treated mice (each sample pooled from single‐nuclei suspensions of 3–4 hearts): (A) t‐SNE visualization identified 8 distinct cell populations—smooth muscle cells (SMCs), pericytes (PCs), NK/T cells, neuronal cells, macrophages (Macs), fibroblasts (FBs), endothelial cells (ECs), and cardiomyocytes (CMs); (B) Dot plot illustrating *Parp7* expression across these cell types. (C,D) Single‐cell RNA sequencing (scRNA‐seq) was conducted on myocardial tissues from LPS‐treated mice (each sample generated by pooling single‐cell suspensions from 3 to 4 hearts): (C) t‐SNE plot revealing seven major cell types, including NK/T cells, B cells, SMCs, PCs, Macs, FBs, and ECs; (D) Dot plot displaying *Parp7* expression patterns within these populations. (E,F) snRNA‐seq was performed on cardiac tissues from patients with heart failure (HF): (E) t‐SNE visualization identified 6 distinct cell populations—SMCs, PCs, Macs, FBs, ECs, and CMs; (F) dot plot illustrating *PARP7 e*xpression across these cell types. (G,H) scRNA‐seq was conducted on myocardial tissues from patients with HF: (G) t‐SNE plot revealing 7 major cell types, including T cells, SMCs, PCs, Macs, FBs, ECs, and B cells; (H) dot plot displaying *PARP7* expression patterns within these populations. (I) The *Parp7* mRNA levels in CMs, FBs, bone marrow‐derived macrophages (BMDMs), and ECs were measured by RT‐qPCR following LPS treatment (10 µg/mL, 24 h, *n* = 8). (J) PARP7 protein levels in BMDMs following LPS treatment were assessed by western blot analysis (*n* = 6). Data are presented as mean ± SEM; **p < 0.01, ***p < 0.001, ns = no significance.

To further elucidate the clinical relevance of PARP7, we conducted snRNA‐seq and scRNA‐seq on myocardial tissues from human failing heart samples. Compared with the murine snRNA‐seq data, human *PARP7* was predominantly upregulated in cardiomyocytes and macrophages, with higher expression levels observed in macrophages (Figure 3E,F). In the human scRNA‐seq dataset, a broader range of cell types exhibited increased PARP7 expression; however, macrophages still displayed the highest overall abundance (Figure 3G,H). Subsequently, we validated the above findings using RT‐qPCR and confirmed that *Parp7* expression was significantly upregulated in both macrophages and cardiomyocytes, with a more pronounced increase observed in macrophages (Figure 3I). Consistent with the mRNA data, the upregulation of PARP7 at the protein level was also detected in BMDMs in a time‐dependent manner (Figure 3J). Overall, through integrated multi‐omics analysis, we demonstrate that PARP7 is predominantly upregulated in macrophages during septic cardiomyopathy.

### 3.4 Macrophage‐Specific Knockdown of PARP7 Aggravates Septic Cardiomyopathy In Vivo

We constructed an AAV9‐*F4/80*‐shPARP7 vector to achieve macrophage‐specific PARP7 knockdown and validated its functional effects in the LPS‐induced septic cardiomyopathy model (Figure 4A and Figure S2A). Our results showed that LPS significantly elevated the serum levels of the myocardial injury markers CK‐MB (Figure 4B) and cTnT (Figure 4C), and these elevations were further exacerbated in mice with macrophage‐specific PARP7 knockdown (Figure 4B,C). Moreover, macrophage‐specific PARP7 knockdown aggravated LPS‐induced cardiac dysfunction (Figure 4D–F). Histopathological analysis via H&E staining revealed that PARP7 knockdown exacerbated LPS‐induced disruption of myofiber architecture (Figure 4G). Immunohistochemical analysis showed that myocardial tissues from AAV9‐*F4/80*‐shPARP7‐injected mice exhibited markedly enhanced LPS‐induced inflammatory cell infiltration, particularly of F4/80^+^ and CD11b^+^ macrophages (Figure 4H–K). Furthermore, quantitative analysis of inflammatory gene expression in myocardial tissues indicated that macrophage‐specific PARP7 knockdown significantly potentiated the LPS‐induced upregulation of *Ifn‐β*, *Tnf‐α*, *Il‐6* and *Il‐1β* (Figure 4L–O). Collectively, these data confirm that macrophage‐specific PARP7 knockdown exacerbates LPS‐induced septic cardiomyopathy.

**FIGURE 4:**
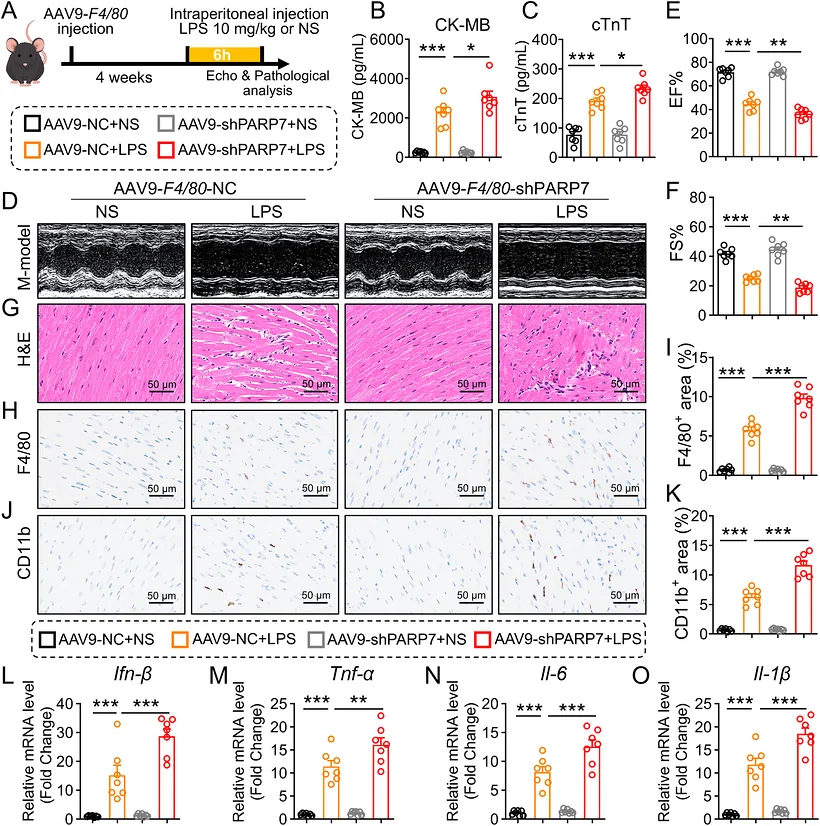
Macrophage‐specific knockdown of PARP7 aggravates LPS‐induced septic cardiomyopathy in mice. (A) Schematic representation of the experimental design: Mice were administered AAV9‐*F4/80*‐vector or AAV9‐*F4/80*‐shPARP7 via tail vein injection at a total dose of 2 × 10^11^ viral genomes (vg). Four weeks post‐injection, mice were intraperitoneally injected with either normal saline (NS) or lipopolysaccharide (LPS, 10 mg/kg). Cardiac function was assessed 6 h after LPS administration. (B,C) Serum levels of creatine kinase‐MB (CK‐MB, B) and cardiac troponin T (cTnT, C) in mice injected with AAV9‐*F4/80*‐vector or AAV9‐*F4/80*‐ shPARP7 and challenged with LPS (*n* = 7). (D) Representative M‐mode echocardiographic tracings of the left ventricle. (E,F) Echocardiographic assessment of ejection fraction (EF%, E) and fractional shortening (FS%, F) (*n* = 7). (G) Representative histological images from H&E staining. (H–K) Representative immunohistochemical staining images for F4/80 (H) and CD11b (J), along with quantitative analyses (I, K) (*n* = 7). (L–O) The mRNA expression levels of *Ifn‐β* (L), *Tnf‐α* (M), *Il‐6* (N), and *Il‐1β* (O) in myocardial tissues of mice injected with AAV9‐*F4/80*‐vector or AAV9‐*F4/80*‐shPARP7 and challenged with LPS (*n* = 7). Data are presented as mean ± SEM; *p < 0.05, **p < 0.01, ***p < 0.001.

We further supplemented the septic cardiomyopathy model induced by CLP. Here, we also constructed an AAV9‐*F4/80*‐shPARP7 vector for macrophage‐specific PARP7 knockdown and tested it in the CLP‐induced septic cardiomyopathy model (Figure S3A). Macrophage‐specific PARP7 knockdown reduced survival outcomes in CLP mice (Figure S3B). Moreover, CLP elevated serum CK‐MB and cTnT (Figure S3C,D), cardiac dysfunction (Figure S3E–G), and myofiber disruption (Figure S3H), all of which were worsened by macrophage‐specific PARP7 knockdown. Additionally, macrophage‐specific PARP7 deficiency enhanced CLP‐induced macrophage infiltration (F4/80^+^ and CD11b^+^; Figure S3I–L), and upregulation of *Ifn‐β*, *Tnf‐α*, *Il‐6*, and *Il‐1β* (Figure S3M–P). Based on the CLP model, we conducted a series of functional validation experiments to further support the protective role of macrophage PARP7 in septic cardiomyopathy.

### 3.5 Macrophage‐Specific Overexpression of PARP7 Attenuates Septic Cardiomyopathy in Mice

To evaluate the therapeutic potential of macrophage‐specific PARP7 in septic cardiomyopathy, mice were intravenously injected with an AAV9 vector containing the *Parp7* gene under the control of the *F4/80* promoter (AAV9‐*F4/80*‐*Parp7*) or an empty control vector (AAV9‐*F4/80*‐vector). The macrophage‐specific overexpression of PARP7 in the myocardial tissues of AAV9‐*F4/80*‐*Parp7*‐treated mice was confirmed by western blot analysis and immunofluorescence staining (Figure S4A,B). Four weeks after viral vector administration, mice were challenged with LPS administration (Figure 5A). LPS treatment significantly increased serum levels of cardiac injury markers CK‐MB (Figure 5B) and cTnT (Figure 5C), both of which were reduced in mice with macrophage‐specific PARP7 overexpression. Furthermore, macrophage‐specific overexpression of PARP7 significantly attenuated LPS‐induced cardiac dysfunction (Figure 5D–F). Histopathological evaluation by H&E staining revealed that PARP7 overexpression alleviated LPS‐induced disorganization of cardiac muscle fibers (Figure 5G). Immunohistochemical analysis of myocardial sections showed that LPS‐induced infiltration of F4/80^+^ and CD11b^+^ macrophages were reduced in AAV9‐*F4/80*‐*Parp7*‐injected mice (Figure 5H–K). Additionally, quantitative analysis of inflammatory gene expression in myocardial tissues demonstrated that macrophage‐specific PARP7 overexpression significantly suppressed LPS‐induced upregulation of *Ifn‐β*, *Tnf‐α*, *Il‐6*, and *Il‐1β* (Figure 5L–O). Collectively, these results indicate that macrophage‐specific PARP7 exerts protective effects against LPS‐induced myocardial injury and dysfunction in vivo.

**FIGURE 5:**
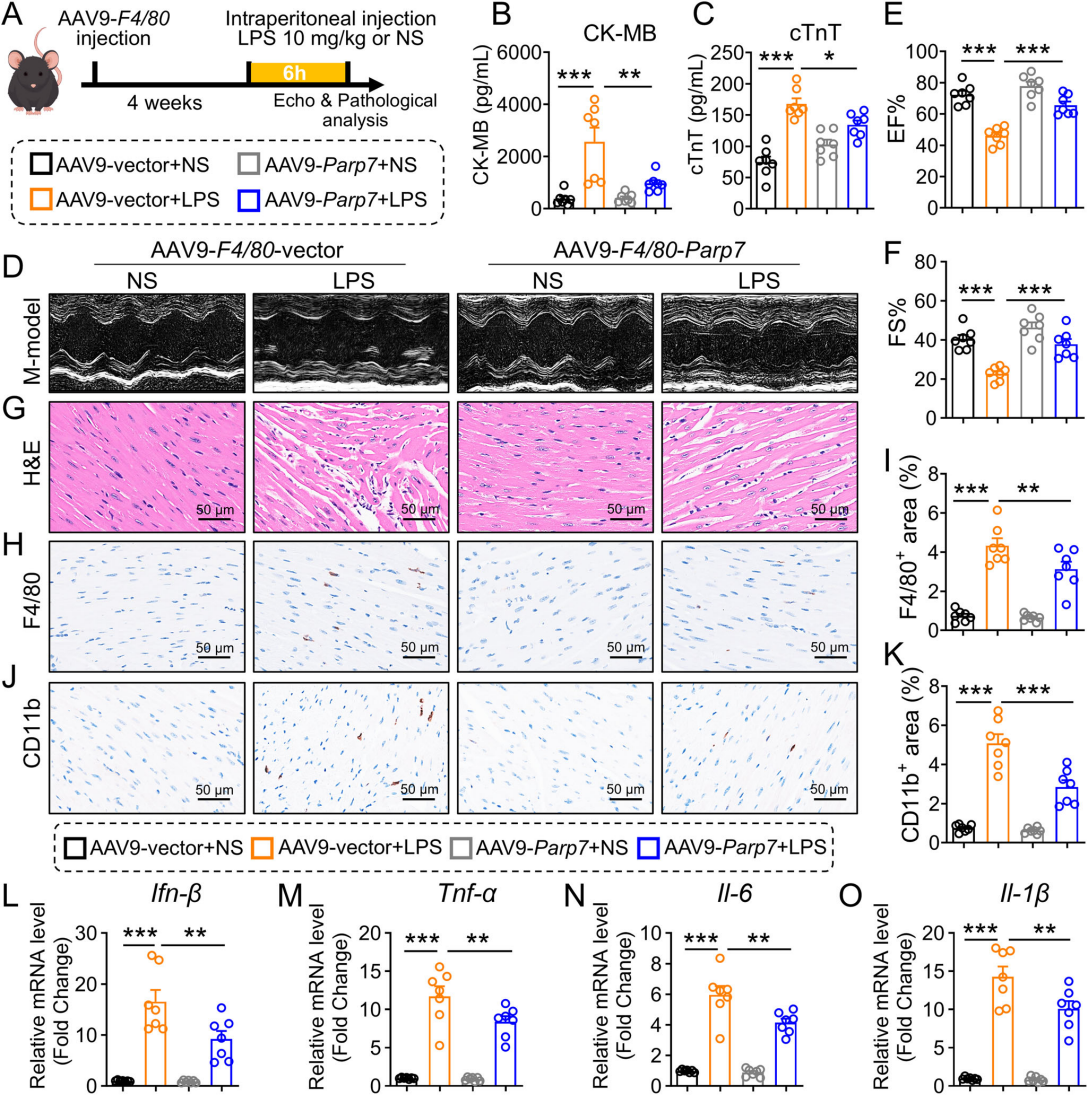
Macrophage‐specific overexpression of PARP7 attenuates LPS‐induced cardiac dysfunction and inflammatory responses in mice. (A) Schematic representation of the experimental design: Mice were administered AAV9‐*F4/80*‐vector or AAV9‐*F4/80*‐*Parp7* via tail vein injection at a total dose of 2 × 10^11^ viral genomes (vg). Four weeks post‐injection, mice were intraperitoneally injected with either normal saline (NS) or lipopolysaccharide (LPS, 10 mg/kg). Cardiac function was assessed 6 h after LPS administration. (B,C) Serum levels of creatine kinase‐MB (CK‐MB, B) and cardiac troponin T (cTnT, C) in mice injected with AAV9‐*F4/80*‐vector or AAV9‐*F4/80*‐*Parp7* and challenged with LPS (*n* = 7). (D) Representative M‐mode echocardiographic tracings of the left ventricle. (E,F) Echocardiographic assessment of ejection fraction (EF%, E) and fractional shortening (FS%, F) (*n* = 7). (G) Representative histological images from H&E staining. (H–K) Representative immunohistochemical staining images for F4/80 (H) and CD11b (J), along with quantitative analyses (I, K) (*n* = 7). (L–O) The mRNA expression levels of *Ifn‐β* (L), *Tnf‐α* (M), *Il‐6* (N), and *Il‐1β* (O) in myocardial tissues of mice injected with AAV9‐*F4/80*‐vector or AAV9‐*F4/80*‐*Parp7* and challenged with LPS (*n* = 7). Data are presented as mean ± SEM; *p < 0.05, **p < 0.01, ***p < 0.001.

### 3.6 Macrophage‐Specific PARP7 Suppresses Inflammatory Responses During Septic Cardiomyopathy

As noted, PARP7 expression was highest in macrophages among all cardiac cell populations, and the upregulation of *Parp7* mRNA in macrophages following LPS treatment was the most pronounced. To investigate the functional role of macrophage‐specific PARP7 in septic cardiomyopathy, we performed snRNA‐seq on hearts from LPS‐treated WT or *Parp7* ^−/−^ mice. A total of 20045 nuclei from two experimental groups were analyzed, and major cardiac cell types were identified, including CMs, ECs, FBs, Macs, PCs, and SMCs (Figure 6A). To identify functionally relevant biological processes, Gene Ontology (GO) enrichment analysis was conducted, revealing that differentially expressed genes (DEGs) in macrophages were significantly enriched in multiple inflammation‐ and immune‐related biological processes. Representative terms included cellular response to LPS, cellular response to IFN‐β, and inflammatory response (Figure 6B). The heat map illustrates the DEGs enriched in the aforementioned inflammation‐ and immune‐related biological processes in macrophages. *Parp7* deficiency further upregulated the expression of multiple pro‐inflammatory genes, including *Il1b*, *Il6*, *Ifnb*, and *Tnf*, a finding consistently supported by our in vivo experimental results (Figure 6C). Immune cells communicate with cardiac parenchymal cells, such as fibroblasts and cardiomyocytes, during heart failure [21]. To investigate the potential interactions between macrophages and other cardiac cell types mediated by PARP7, we performed CellChat analysis on snRNA‐seq data and found that *Parp7* knockout enhanced the potential for intercellular communication between macrophages and other cardiac cell types (Figure 6D). These interactions were characterized by the type, number, and strength of reciprocal receptor‐ligand pairs. Considering that the absence of PARP7 transforms macrophages into central drivers of the inflammatory regulatory network, we employed a mouse cytokine array to investigate PARP7‐mediated cytokine secretion in macrophages. As shown in Figure 6E, *Parp7* knockout increases the secretion of multiple cytokines in LPS‐treated BMDMs, including IFN, IL‐1β, IL‐6, and TNF‐α, a finding consistently supported by our in vivo experimental results and snRNA‐seq data.

**FIGURE 6:**
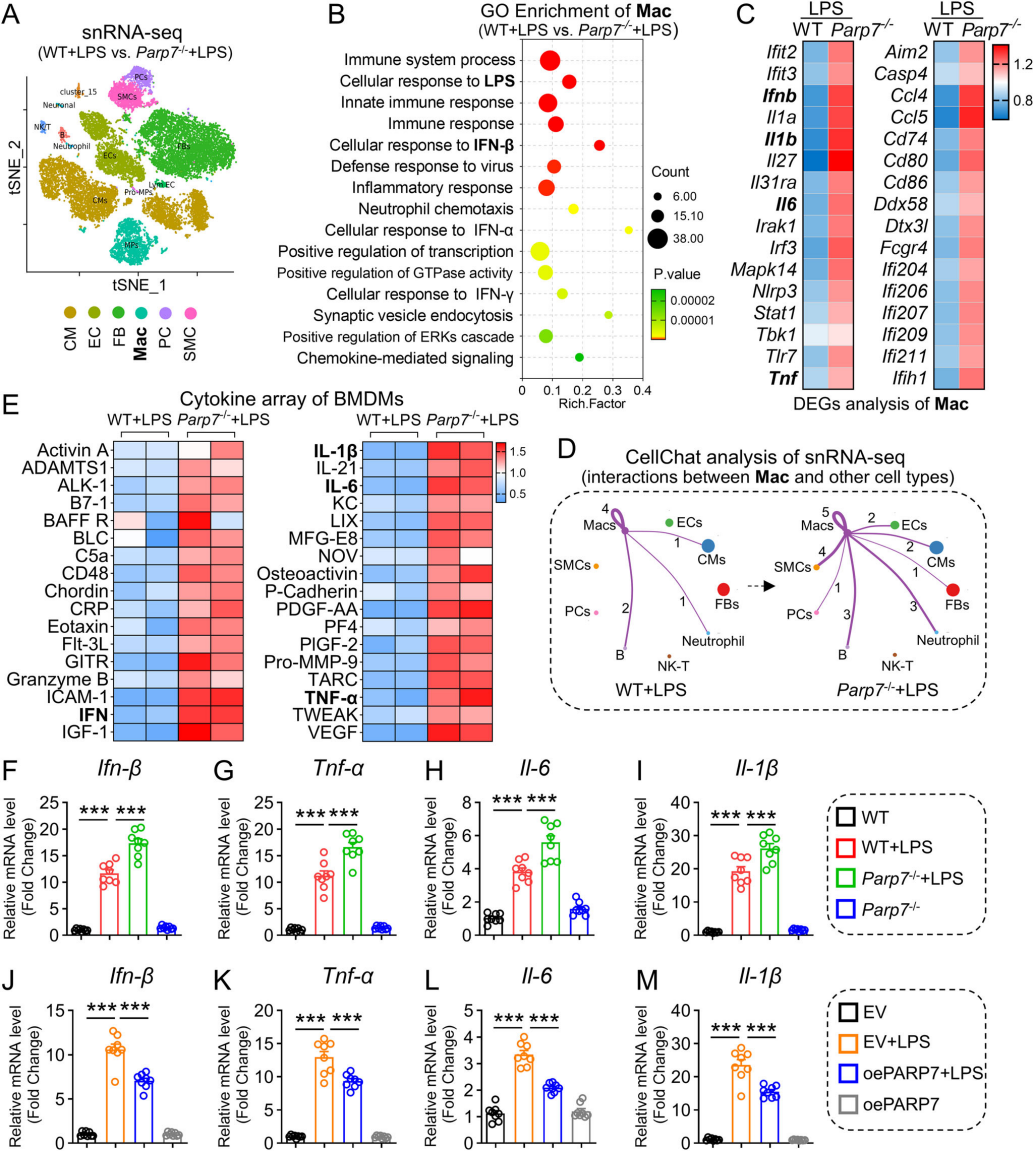
Single‐nucleus RNA sequencing reveals that macrophage PARP7 suppresses inflammatory responses during septic cardiomyopathy. (A) Single‐nucleus RNA sequencing (snRNA‐seq) was performed on cardiac tissues from wild‐type (WT) or *Parp7* ^−/−^ mice treated with LPS (10 mg/kg, 6 h); each sample was pooled from single‐nuclei suspensions of 3–4 hearts. The t‐SNE plot displays the major cell populations, including cardiomyocytes (CMs), endothelial cells (ECs), fibroblasts (FBs), macrophages (Macs), pericytes (PCs), and smooth muscle cells (SMCs). (B) Gene Ontology (GO) enrichment analysis of macrophages (Macs) in cardiac tissues from WT or *Parp7* ^−/−^ mice following LPS challenge. (C) Heatmaps showing the differential expression gene (DEG) analysis of genes associated with signaling pathways identified by GO enrichment analysis. (D) Network diagram illustrating the potential interactions between macrophages and other cardiac cell types mediated by PARP7, based on CellChat analysis of snRNA‐seq data (the numerical values on the connection lines represent interaction strength). (E) Heatmap showing supernatant cytokine levels detected by mouse cytokine antibody array analysis. Bone marrow‐derived macrophages (BMDMs) were treated with LPS (10 µg/mL, 24 h), and the supernatant was collected for cytokine profiling using the mouse cytokine array Q4000. (F–I) The mRNA levels of *Ifn‐β* (F), *Tnf‐α* (G), *Il‐6* (H), and *Il‐1β* (I) in BMDMs isolated from WT or *Parp7* ^−/−^ mice following LPS stimulation (*n* = 8). (J–M) The mRNA levels of *Ifn‐β* (J), *Tnf‐α* (K), *Il‐6* (L), and *Il‐1β* (M) in LPS‐induced BMDMs transfected with empty vector (EV) or Flag‐PARP7 plasmids (oePARP7) (*n* = 8). Data are presented as mean ± SEM; ***p < 0.001.

Next, we validated the snRNA‐seq and cytokine array findings using RT‐qPCR. *Parp7* deficiency (Figure S5A,B) significantly exacerbated LPS‐induced expression of pro‐inflammatory cytokines in BMDMs, including *Ifn‐β*, *Tnf‐α*, *Il‐6* and *Il‐1β* (Figure 6F–I), whereas PARP7 overexpression (oePARP7; Figure S5C) in LPS‐treated BMDMs markedly suppressed their induction (Figure 6J–M). Consistent with this cytokine profile, dual immunofluorescence staining for iNOS (an M1 marker) and CD206 (an M2 marker) revealed that *Parp7* knockout promoted M1 polarization and concurrently impaired M2 accumulation (Figure S5D,E). Furthermore, flow cytometric analysis and snRNA‐seq confirmed *Parp7* deficiency increased proportion of M1 macrophages from LPS‐treated *Parp7^−/−^* mice relative to LPS‐treated WT mice (Figure S5F–J). To assess translational relevance, we extended these analyses to human macrophages derived from THP‐1 cells. THP‐1 monocytes were differentiated into macrophages using PMA, followed by PARP7 overexpression prior to LPS challenge. As observed in murine BMDMs, PARP7 overexpression (Figure S6A) significantly attenuated LPS‐induced mRNA expression of *Ifn‐β*, *Tnf‐α*, *Il‐6*, and *Il‐1β* in human THP‐1 macrophages (Figure S6B–E). Collectively, these cross‐species results demonstrate that PARP7 functions as a conserved endogenous brake on inflammatory signaling in macrophages.

### 3.7 PARP7 Interacts With TBK1 to Mediate its ADP‐Ribosylation, Thereby Suppressing TBK1‐Driven Inflammatory Response in Macrophages During Septic Cardiomyopathy

PARP7, as a mono‐ADP‐ribose transferase, primarily exerts its biological functions by binding to specific substrates and regulating their ADP‐ribosylation [12]. Recently, emerging evidence has indicated that TBK1 is a key substrate of PARP7, with PARP7 interacting with TBK1 to negatively regulate IFN‐I signaling [14, 22]. In this study, we have demonstrated that PARP7 suppresses LPS‐induced multiple cytokines from macrophages through the negative regulation of IFN‐I signaling. Therefore, we hypothesized that PARP7 may also target TBK1 as a crucial substrate in septic cardiomyopathy. First, we detected the endogenous interaction between PARP7 and TBK1 in LPS‐treated BMDMs using proximity ligation assay (PLA) (Figure 7A) and co‐IP (Figure 7B). Subsequently, we confirmed this endogenous binding between PARP7 and TBK1 in myocardial tissues of septic mice (Figure 7C). Next, the physical binding of exogenously expressed PARP7 and TBK1 was observed in NIH/3T3 cells co‐transfecting Flag‐PARP7 and His‐TBK1 plasmids (Figure 7D). The PARP7 protein contains three main domains: a cysteine‐cysteine‐cysteine‐histidine (CCCH) zinc finger domain responsible for DNA/RNA binding, a tryptophan‐tryptophan‐glutamate (WWE) domain that mediates protein‐protein or iso‐ADP‐ribose interactions, and a conserved ADP‐ribosyl transferase catalytic (PARP) domain that exhibits ADP‐ribosyl transferase activity [22, 23] (Figure 7E). To identify the domain required for the interaction between PARP7 and TBK1, we co‐transfected NIH/3T3 cells with His‐TBK1 and either full‐length Flag‐PARP7 (PARP7‐WT) or its deletion mutants lacking the CCCH domain (△CCCH), the WWE domain (△WWE), or the PARP domain (△PARP). As shown in Figure 7F, TBK1 interacted with PARP7‐WT, PARP7‐△CCCH, and PARP7‐△PARP, but not with PARP7‐△WWE, indicating that the WWE domain of PARP7 is essential for its interaction with TBK1.

**FIGURE 7:**
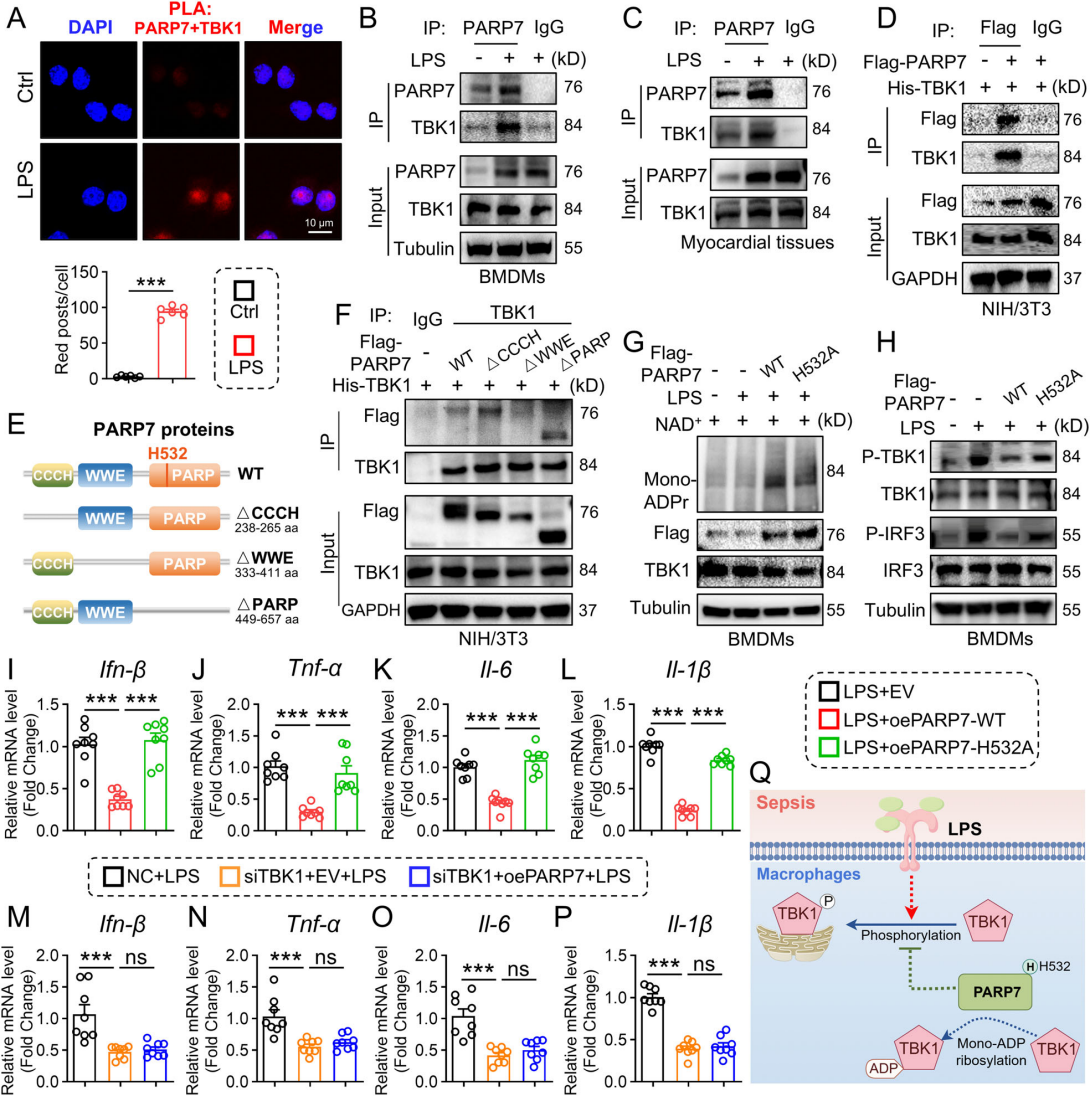
PARP7 interacts with TBK1 to mediate its ADP‐ribosylation, thereby suppressing TBK1‐driven inflammatory response in macrophages during septic cardiomyopathy. (A) Representative images from proximity ligation assay (PLA) showing endogenous interaction between PARP7 and TBK1 in BMDMs treated with LPS (10 µg/mL, 24 h), along with quantitative analysis of red fluorescent signals per cell (*n* = 6). (B,C) Co‐immunoprecipitation (Co‐IP) analysis of PARP7 and TBK1 in LPS‐treated BMDMs (B) and myocardial tissues from septic mice (C). Endogenous PARP7 was immunoprecipitated using an anti‐PARP7 antibody. (D) Co‐IP of Flag‐PARP7 and His‐TBK1 in NIH/3T3 cells co‐transfected with Flag‐PARP7 and His‐TBK1. Exogenous Flag‐PARP7 was pulled down with an anti‐Flag antibody. (E) Schematic representation of full‐length PARP7 (wild‐type, WT) and its deletion mutants: ∆CCCH (lacking the CCCH domain), ∆WWE (lacking the WWE domain), and ∆PARP (lacking the PARP domain). The catalytic residue histidine 532 (H532) within the PARP domain confers mono‐ADP‐ribosyltransferase activity. (F) NIH/3T3 cells were co‐transfected with His‐TBK1 and either Flag‐PARP7‐WT, ∆CCCH, ∆WWE, or ∆PARP mutants. Cell lysates were subjected to immunoprecipitation with an anti‐TBK1 antibody to determine the PARP7 domain required for interaction. (G) BMDMs were transfected with Flag‐PARP7‐WT or Flag‐PARP7‐H532A (carrying a histidine‐to‐alanine substitution at position 532) and stimulated with LPS, followed by treatment with 10 µm NAD^+^. Western blot analysis was performed to assess mono‐ADP‐ribosylation levels, as well as the expression of Flag and TBK1 in cell lysates. (H) BMDMs were transfected with Flag‐PARP7‐WT or Flag‐PARP7‐H532A and stimulated with LPS. Western blot analysis was conducted to evaluate phosphorylation levels of TBK1 and IRF3. (I–L) The mRNA levels of *Ifn‐β* (I), *Tnf‐α* (J), *Il‐6* (K), and *Il‐1β* (L) in LPS‐stimulated BMDMs overexpressing Flag‐PARP7‐WT (oePARP7‐WT) or Flag‐PARP7‐H532A (oePARP7‐H532A) (*n* = 8). (M–P) The mRNA levels of *Ifn‐β* (M), *Tnf‐α* (N), *Il‐6* (O), or *Il‐1β* (P) in BMDMs transfected with siRNA targeting TBK1 (siTBK1) prior to Flag‐PARP7 plasmid transfection (oePARP7), followed by LPS stimulation (*n* = 8). (Q) Schematic illustration showing that PARP7 interacts with TBK1 to mediate its ADP‐ribosylation, thereby suppressing TBK1 phosphorylation in macrophages upon LPS stimulation. Data are presented as mean ± SEM; ***p < 0.001, ns = no significance.

The catalytic residue histidine 532 (H532) within the PARP domain is responsible for conferring mono‐ADP‐ribosyl transferase activity [12] (Figure 7E). A PARP7‐H532A mutant, in which histidine at position 532 is substituted with alanine, was generated. As shown in Figure 7G, LPS‐induced ADP‐ribosylation of TBK1 was detected in cells expressing PARP7‐WT and was inhibited in the presence of the catalytically inactive PARP7‐H532A mutant in BDMDs, indicating that the ADP‐ribosylation of TBK1 depends on the catalytic activity of H532 in PARP7. TBK1 is a central kinase in the innate immune response that phosphorylates the transcription factors IRF3 and NF‐κB subunit p65, thereby promoting their nuclear translocation and enabling transcriptional activation of pro‐inflammatory cytokine and IFN‐I genes [24]. We examined the impact of PARP7 on the activation kinetics of the TBK1‐IRF3/NF‐κB signaling axes and found that PARP7 overexpression significantly suppressed LPS‐induced phosphorylation of TBK1 and its downstream effectors IRF3 (Figure 7H) and NF‐κB subunit p65 (Figure S7A) in BMDMs, whereas this inhibitory effect was attenuated by the catalytically inactive PARP7‐H532A mutant (Figure 7H and Figure S7A). Moreover, PARP7 overexpression significantly reduced the mRNA expression of TBK1‐dependent inflammatory cytokines in LPS‐stimulated BMDMs (Figure 7I–L), including *Ifn‐β*, *Tnf‐α*, *Il‐6* and *Il‐1β*, an effect that was largely reversed by the PARP7‐H532A mutant (Figure 7I–L). To validate that TBK1 is the primary substrate through which PARP7 modulates inflammatory responses in BMDMs, we performed TBK1 knockdown (siTBK1, Figure S7B) in combination with PARP7 overexpression prior to LPS stimulation. As expected, TBK1 knockdown substantially suppressed LPS‐induced upregulation of inflammatory cytokines (Figure 7M–P). Notably, PARP7 overexpression did not further reduce cytokine production in TBK1‐knockdown BMDMs (Figure 7M–P), demonstrating that TBK1 is essential for PARP7‐mediated suppression of the inflammatory response. Overall, PARP7 interacts with TBK1 to mediate its ADP‐ribosylation, thereby inhibiting TBK1‐driven inflammatory signaling in macrophages during septic cardiomyopathy.

## 4 Discussion

PARPs constitute a family of 18 enzymes that catalyze the transfer of ADP‐ribose to target substrates, a reversible post‐translational modification referred to as ADP‐ribosylation [5]. Several PARP family members, including PARP1 and PARP2, have been implicated in various forms of myocardial injury through this modification [7, 8, 9]. Our previous studies have also demonstrated that PARP1 contributes to ventricular remodeling by regulating cardiomyocyte parthanatos [11]. These findings suggest that the PARP family may serve as a valuable molecular resource for identifying novel therapeutic targets in septic cardiomyopathy. In this study, comprehensive analysis of PARP family gene expression in LPS‐treated myocardial tissues revealed that PARP7 is potentially associated with septic cardiomyopathy. PARP7, a mono‐ADP‐ribosyltransferase, was initially identified as a target gene of the aryl hydrocarbon receptor (AHR) activated by dioxin exposure [12]. More recently, PARP7 has been recognized as a key negative regulator of IFN‐I signaling and plays a central role in tumor immune evasion [14]. Atamparib (RBN‐2397), a first‐in‐class selective PARP7 inhibitor, was approved in 2025 [23]. Its therapeutic mechanism involves targeting PARP7 in tumor cells, resulting in direct suppression of tumor cell proliferation and reactivation of IFN‐I signaling [25]. This dual action promotes both innate and adaptive anti‐tumor immune responses, offering a promising therapeutic strategy for advanced solid tumors. However, the widespread expression of PARP7 across multiple organs suggests additional biological functions that likely extend beyond immune regulation [12]. Our in vivo experiments demonstrated that PARP7 exerts a cardioprotective effect against LPS‐induced cardiac dysfunction, histopathological injury, inflammatory cell infiltration, and excessive cytokine production. These findings provide important evidence regarding potential complications in the clinical use of PARP7 inhibitors—specifically, that such inhibitors may induce cardiotoxicity, at least in the context of sepsis‐associated cardiac inflammation.

Macrophages, as key sentinels and regulators of innate immunity, shape the progression of sepsis by modulating their activation state and the specific type of interferon produced in response to danger signals, including pathogens, necrotic cells, and immune complexes [4]. In cardiac tissues, macrophages represent the predominant immune cell population, accounting for 5%‐10% of non‐myocyte cells [26]. Histological and immunohistochemical analyses of myocardial samples from patients with prolonged severe sepsis or septic shock have revealed extensive infiltration of activated macrophages within the myocardial interstitium, diffusely distributed throughout the heart, accompanied by marked upregulation of multiple inflammatory factors [27]. At the molecular level, TLR4 on macrophages binds LPS, triggering downstream signaling pathways such as NF‐κB, which enhances the production of pro‐inflammatory mediators and thereby exacerbates myocardial inflammation during septic cardiomyopathy [28]. In this study, integrated single‐nucleus and single‐cell RNA sequencing analyses reveal that PARP7 is predominantly upregulated in cardiac macrophages of LPS‐treated mice. Subsequently, we validate the cardioprotective role of macrophage‐specific PARP7 in septic mice using an AAV9‐mediated delivery system. These findings not only expand and refine the molecular mechanistic network underlying macrophage‐mediated regulation of septic cardiomyopathy but also provide a significant targeted strategy for macrophage‐oriented therapy in clinical myocardial injury.

Unlike other members of the PARP family, PARP7 exhibits distinct substrate specificity by modifying unique acceptor sites [12]. It preferentially modifies a range of substrate proteins, including AHR, TBK1, HIF‐1α, and FRA1, thereby regulating their stability and activity [12]. Notably, the PARP7‐TBK1 interaction has become a central focus in the development of selective PARP7 inhibitors. PARP7 binds to TBK1 and suppresses its activity through ADP‐ribosylation, leading to inhibition of AHR‐mediated IFN‐I production during viral infection [29]. Independently, Gozgit et al. demonstrated that PARP7 acts as a negative regulator in cytosolic nucleic acid sensing in a TBK1‐dependent manner, showing that PARP7 inhibition elicits both cancer cell‐autonomous effects and antitumor immunity via enhanced IFN signaling [14]. TBK1, as a core component of innate immune sensing pathways, plays a pivotal role in multiple cardiac pathologies, including heart failure [30], doxorubicin‐induced cardiotoxicity [31], viral myocarditis [32], myocardial infarction [33], and diabetic cardiomyopathy [34]. In this study, we demonstrate that PARP7 suppresses LPS‐induced cytokine production in macrophages by negatively regulating IFN‐I signaling. Based on these findings, we hypothesized that TBK1 may serve as a key substrate of PARP7 in the context of septic cardiomyopathy. Our data confirm that PARP7 directly interacts with TBK1 and catalyzes its ADP‐ribosylation via the catalytic residue H532, resulting in suppression of downstream inflammatory signaling in LPS‐stimulated macrophages.

Several TBK1 inhibitors, including Amlexanox, BX795, and derivatives of CYT387, have advanced into preclinical or early clinical development, primarily in the field of oncology [35]. Notably, cardiac toxicity associated with these inhibitors has emerged as a critical safety concern, warranting active monitoring in both preclinical and clinical studies [35, 36]. Previous evidence has demonstrated that cardiomyocyte‐specific deletion of TBK1 exacerbates chronic doxorubicin‐induced cardiotoxicity by impairing mitophagy [31]. Collectively, these findings suggest that TBK1 may exert cardioprotective effects, particularly within cardiomyocytes, at least under specific pathological conditions such as chronic myocardial injury. In contrast, our results reveal that macrophage‐specific PARP7 alleviates septic cardiomyopathy by suppressing TBK1 activity, highlighting two important implications: first, activation of TBK1 in macrophages may promote cardiac injury; second, this detrimental effect appears to be more pronounced in acute inflammatory settings. Given that direct inhibition of TBK1 carries risks of widespread off‐target effects and systemic toxicity, selectively targeting TBK1 in macrophages offers a promising alternative strategy. Therefore, developing a macrophage‐specific PARP7‐TBK1 signaling modulation system could enable temporally and spatially precise therapeutic intervention.

In summary, this study reveals a significant molecular mechanism underlying LPS‐induced septic cardiomyopathy. As a multifunctional ADP‐ribosyl transferase, PARP7 directly interacts with TBK1 and catalyzes its ADP‐ribosylation, thereby suppressing the TBK1‐mediated downstream inflammatory response and ultimately attenuating cardiac dysfunction in septic cardiomyopathy. Our findings establish a PARP7‐TBK1 regulatory axis in this condition and highlight the therapeutic potential of targeting macrophage‐specific PARP7 for the treatment of septic cardiomyopathy.

## Author Contributions

J.H., X.S., J.X., and L.W. contributed to the literature search and study design. X.S., L.W., X.Z., S.Z., H.X., X.H., L.L., Y.L., and K.X. carried out the experiments. X.S., J.H., and J.X. contributed to data collection and analysis. J.H., X.S., and J.X. participated in the drafting of the article. J.H., X.S., L.W., and X.Z. contributed essential reagents or tools.

## Funding

This study was supported by Natural Science Foundation of Zhejiang Province (Nos. ZCLMS26H1501 to X.W. Shi, ZCLMS25H1503 to J.B. Han, ZCLQN25H0203 to X. Zhong), the National Natural Science Foundation of China (Nos. 82470384 to J.B. Han, 82400396 to X.W. Shi), the grants from Zhejiang Provincial Health Bureau Science Foundation, Hangzhou, China (Nos. 2025KY363 to X.W. Shi, 2025KY355 to X. Zhong), and the China Postdoctoral Science Foundation (No. 2025M781377 to L.T. Wang).

## Conflicts of Interest

The authors declare no conflicts of interest.

## Supporting information




**Supporting File**: advs77404‐sup‐0001‐SuppMat.docx.

## Data Availability

Data sharing not applicable to this article as no datasets were generated or analyzed during the current study.
