# Supplementary material for: Macrophage PARP7 Alleviates Septic Cardiomyopathy by Interacting With TBK1 and Suppressing TBK1‐Driven Inflammatory Response
Source: Adv Sci (Weinh). 2026 Aug 24;13(63):e77404. doi: 10.1002/advs.77404 (PMC13502346; doi:10.1002/advs.77404)
Supplement: Supplementary file 1 [file ADVS-13-e77404-s001.docx]

***Supplemental Material***

**Macrophage PARP7 alleviates** **septic cardiomyopathy by interacting with TBK1 and suppressing TBK1-driven inflammatory response**

Supplementary Information:

1. Supplementary Figure S1-7
2. Supplementary Table S1

***Supplementary Figures:***

**Figure S1. (A)** Genotyping of mice was performed by PCR using primers specific for the wild-type (WT) and *Parrp7*^−/−^ alleles. The amplified PCR products yielded fragments of 271 bp for the WT allele and 302 bp for the *Parrp7*^−/−^ allele, respectively. **(B)** The knockout efficiency of PARP7 in myocardial tissues from WT or *Parp7*^-/-^ mice verified by western blotting. Data are presented as mean ± SEM; ****P* < 0.001; n=6.

**Figure S2. (A)** The knockdown efficiency of PARP7 in myocardial tissues from AAV9-*F4/80*-NC and AAV9-*F4/80*-shPARP7 mice verified by western blotting. Data are presented as mean ± SEM; ****P* < 0.001; n=6.

**Figure S3. Macrophage-specific knockdown of PARP7 aggravates CLP-induced septic cardiomyopathy in mice. (A)** Experimental workflow for AAV9-*F4/80*-NC and AAV9-*F4/80*-shPARP7 mice underwent either a sham operation or a cecal ligation and puncture (CLP). **(B)** Survival curves of AAV9-*F4/80*-NC and AAV9-*F4/80*-shPARP7 mice after sham or CLP surgery (n = 12). **(C-D)** Serum levels of creatine kinase-MB (CK-MB, **C**) and cardiac troponin T (cTnT, **D**) in AAV9-*F4/80*-NC and AAV9-*F4/80*-shPARP7 mice following CLP treatment (n = 7). **(E)** Representative M-mode echocardiographic images of the left ventricle. **(F-G)** Echocardiographic assessment of ejection fraction (EF%, **F**) and fractional shortening (FS%, **G**) in mice (n = 7). **(H)** Representative hematoxylin-eosin (H&E) staining images. **(I-L)** Representative immunohistochemical staining images for F4/80 (**I**) and CD11b (**K**), along with corresponding quantitative results (**J, L**) (n = 7). **(M-P)** The mRNA expression levels of *Ifn-β* (**M**), *Tnf-α* (**N**), *Il-6* (**O**) and *Il-1β* (**P**) in myocardial tissues from AAV9-*F4/80*-NC and AAV9-*F4/80*-shPARP7 mice following CLP treatment (n = 7). Data are presented as mean ± SEM; ***P* < 0.01, ****P* < 0.001.

**Figure S4. (A)** The overexpression efficiency of AAV9-*F4/80*-*Parp7* in bone marrow-derived macrophages (BMDMs) from mice verified by western blotting (n=6). **(B)** Representative immunofluorescence staining images of heart tissues from mice treated with AAV9-*F4/80*-*Parp7*, showing immunoreactivity to F4/80 (green) and Flag-PARP7 (red). Cell nuclei were stained with DAPI (blue). Merged images indicate co-localization (yellow). Data are presented as mean ± SEM; ***P* < 0.01.

**Figure S5. (A)** Western blot detection and quantitative analysis of PARP7 protein levels in bone marrow-derived macrophages (BMDMs) isolated from WT and *Parp7*^−/−^ mice (n = 6). **(B)** The knockout efficiency of *Parp7* in BMDMs isolated from WT or *Parp7*^-/-^ mice verified by RT-qPCR (n = 8). **(C)** The overexpression efficiency of Flag-PARP7 plasmids (oePARP7) in BMDMs verified by western blotting (n = 6). **(D-E)** Heart tissue sections were stained for the M1 macrophage biomarker iNOS and M2 macrophage marker CD206, followed by DAPI nuclear counterstaining. Representative staining images and quantification of positive cells are shown (n = 6). **(F-H)** Flow cytometric analysis of the heart validated the ratio of M1 macrophage (**G**) and M2 macrophage (**H**) from LPS-treated WT and *Parp7*^-/-^ mice (n =3). **(I)** tSNE plot of monocyte/macrophage clusters in hearts from LPS-treated WT and *Parp7*^-/-^ mice. **(J)** Bar chart shows the proportions of M1 macrophage, M2 macrophage, and monocytes in hearts from LPS-treated WT and *Parp7*^-/-^ mice. Data are presented as mean ± SEM; **P* < 0.05, ****P* < 0.001.

.

**Figure S6. (A)** The overexpression efficiency of Flag-PARP7 plasmids (oePARP7) in THP-1 cells verified by western blotting (n = 6). **(B-E)** The mRNA levels of *Ifn-β* (**B**), *Tnf-α* (**C**), *Il-6* (**D**) and *Il-1β* (**E**) in LPS-induced THP-1 cells transfected with empty vector (EV) or Flag-PARP7 plasmids (oePARP7) (n = 8). Data are presented as mean ± SEM; ****P* < 0.001.

**Figure S7. (A)** BMDMs were transfected with Flag-PARP7-WT or Flag-PARP7-H532A and stimulated with LPS. Western blot analysis was conducted to evaluate phosphorylation levels of NF-κB/p65. **(B)** The knockdown efficiency of siTBK1 in BMDMs verified by western blotting (n=6). Data are presented as mean ± SEM; ****P* < 0.001.

***Supplementary Tables:***

**Table S1. Primers for RT-qPCR**

| **Gene name** | Species | **Sequence (5’ to 3’)** |
| --- | --- | --- |
| *Parp1* | Mouse | F: GCGGAGAAGACATTGGGTGA |
|  |  | R: ACCATCTTCTTGGACAGGCG |
| *Parp2* | Mouse | F: TGCTTTGCCTCTCGCCTAAA |
|  |  | R: TCCCCTTGGTGCTATGCTTG |
| *Parp3* | Mouse | F: TGAACCAGACCAACATCGGC |
|  |  | R: TTCCAGCAGAAGAAGCGACT |
| *Parp4* | Mouse | F: TGACCTCCGTGGAAAGAACG |
|  |  | R: ACAGAGCATACAGGGCAACC |
| *Parp6* | Mouse | F: ACTGACTCCCACCCCTGATT |
|  |  | R: AGTCCACAGCACCTTCCATTT |
| *Parp7* | Mouse | F: CCAACTCTCGGGGTCTGAAA |
|  |  | R: CAGGAACGGAAAAGGGGTCT |
| *Parp8* | Mouse | F: TGCCCCCAAGACATCGAAAA |
|  |  | R: CGGTCTCGGATTGGAATGCT |
| *Parp9* | Mouse | F: CCCCACTGTTGCGTCCTTTA |
|  |  | R: CCGATGCGAAGAGTCATGGT |
| *Parp10* | Mouse | F: GAGCTTGAGACTGGAGGACG |
|  |  | R: CAGCCTCAAGTGTGGGGATT |
| *Parp11* | Mouse | F: TGGGAGAACGTGAACCCTGA |
|  |  | R: TCATTATACTCGTGCGTCTGGT |
| *Parp12* | Mouse | F: CCAACTCTCGGGGTCTGAAA |
|  |  | R: CAGGAACGGAAAAGGGGTCT |
| *Parp14* | Mouse | F: GATTTTGCTGTTGCCGTGGT |
|  |  | R: TGATTTGAGCGGTGGCTGAT |
| *Ifn-β* | Mouse | F: ACTGCCTTTGCCATCCAAGA |
|  |  | R: CACTGTCTGCTGGTGGAGTT |
| *Tnf-α* | Mouse | F: ATAGCTCCCAGAAAAGCAAGC |
|  |  | R: AGTGCCTCTTCTGCCAGTTC |
| *IL-6* | Mouse | F: TAGTCCTTCCTACCCCAATTTCC |
|  |  | R: CGCACTAGGTTTGCCGAGTA |
| *IL-1β* | Mouse | F: GGTGTGTGACGTTCCCATTA |
|  |  | R: ATTGAGGTGGAGAGCTTTCAG |
| *Ifn-β* | Human | F: TGGCACAACAGGTAGTAGGC  R: AGTGGAGAAGCACAACAGGAG |
| *Tnf-α* | Human | F: TATCCTGGGGGACCCAATGT  R: AAAAGAAGGCACAGAGGCCA |
| *IL-6* | Human | F: TGAGGAGACTTGCCTGGTGAA  R: CAGCTCTGGCTTGTTCCTCAC |
| *IL-1β* | Human | F: CTGTACCTGTCCTGCGTGTT  R: GGGAACTGGGCAGACTCAAA |
| *β-Actin* | Mouse | F: CTCTCCCTCACGCCATC  R: ACGCACGATTTCCCTCTC |
| *β-Actin* | Human | F: GTGGCATCCACGAAACTACCTT  R: GGACTCGTCATACTCCTGCTTG |
